# A Rare Presentation of Polyarteritis Nodosa

**DOI:** 10.7759/cureus.21925

**Published:** 2022-02-05

**Authors:** Ciji Robinson, Zarqa Yasin, Parth Patel, Hazem Zebda

**Affiliations:** 1 Internal Medicine, Henry Ford Health System, Jackson, USA; 2 Rheumatology, Henry Ford Health System, Detroit, USA

**Keywords:** biopsy, arterial dissection, rheumatoid vasculitis, segmental arterial mediolysis, polyarteritis nodosom

## Abstract

Polyarteritis nodosa (PAN) is a rare form of necrotizing medium-vessel vasculitis. PAN has the potential for widespread organ involvement, but the skin, renal, neurologic, and musculoskeletal systems are most commonly involved. A definitive diagnosis can be made with a biopsy of an easily accessible organ such as the skin or an involved nerve or muscle. We present a case of a 66-year-old female with no significant past medical history who presented with chest and epigastric pain. She was subsequently found to have computed tomography angiography (CTA) findings consistent with PAN, including areas of arterial narrowing alternating with areas of aneurysmal dilation confined to the mesenteric arteries. A biopsy of the involved arteries was deemed unsafe and ultimately not performed. Her lab findings were remarkable for elevated erythrocyte sedimentation rate (ESR) and C-reactive protein (CRP) and unremarkable for a broad infectious disease workup. Suspicion of PAN was further strengthened by a positive response to, and eventual full recovery on, high-dose steroids alone.

## Introduction

Polyarteritis nodosa (PAN) is most commonly associated with medium-sized vessels, but small vessels may also be involved [1]. PAN is a widespread form of vasculitis and can affect many different organ systems. However, an important characterizing factor for PAN is that it spares the lungs [2]. It is typically seen in adults over 50 years of age, and its prevalence has declined since the standardization of the Hepatitis B virus (HBV) vaccine, as PAN and HBV have been historically associated with each other [2]. PAN has no other significant associations and is most often deemed idiopathic [1]. Findings that should raise clinical suspicion for PAN are computed tomography angiography (CTA) findings remarkable for arterial narrowing alternating with areas of aneurysmal dilation and possibly areas of arterial dissection [3]. Elevated inflammatory markers and a lack of infectious etiology also point towards the diagnosis [3]. Additionally, involved organs typically show areas of ischemia on imaging studies as vascular changes associated with PAN compromise blood flow to end organs [2].

## Case presentation

**Figure 1:**
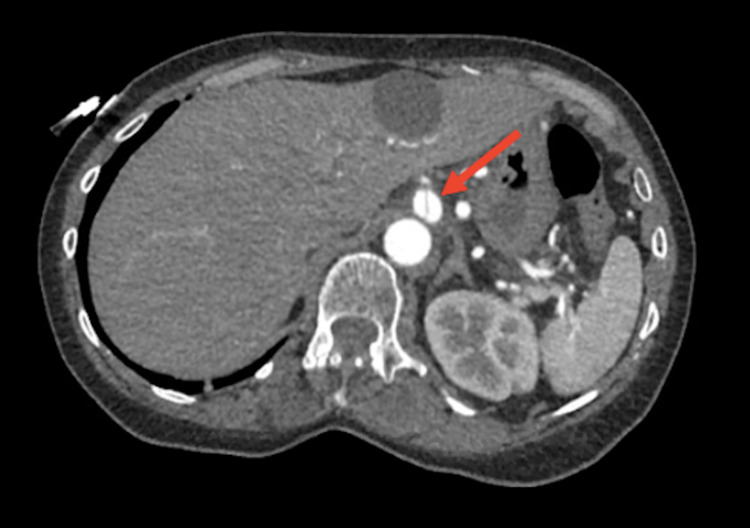
Axial CTA demonstrating celiac artery dissection (arrow) CTA: computed tomography angiography.

**Figure 2:**
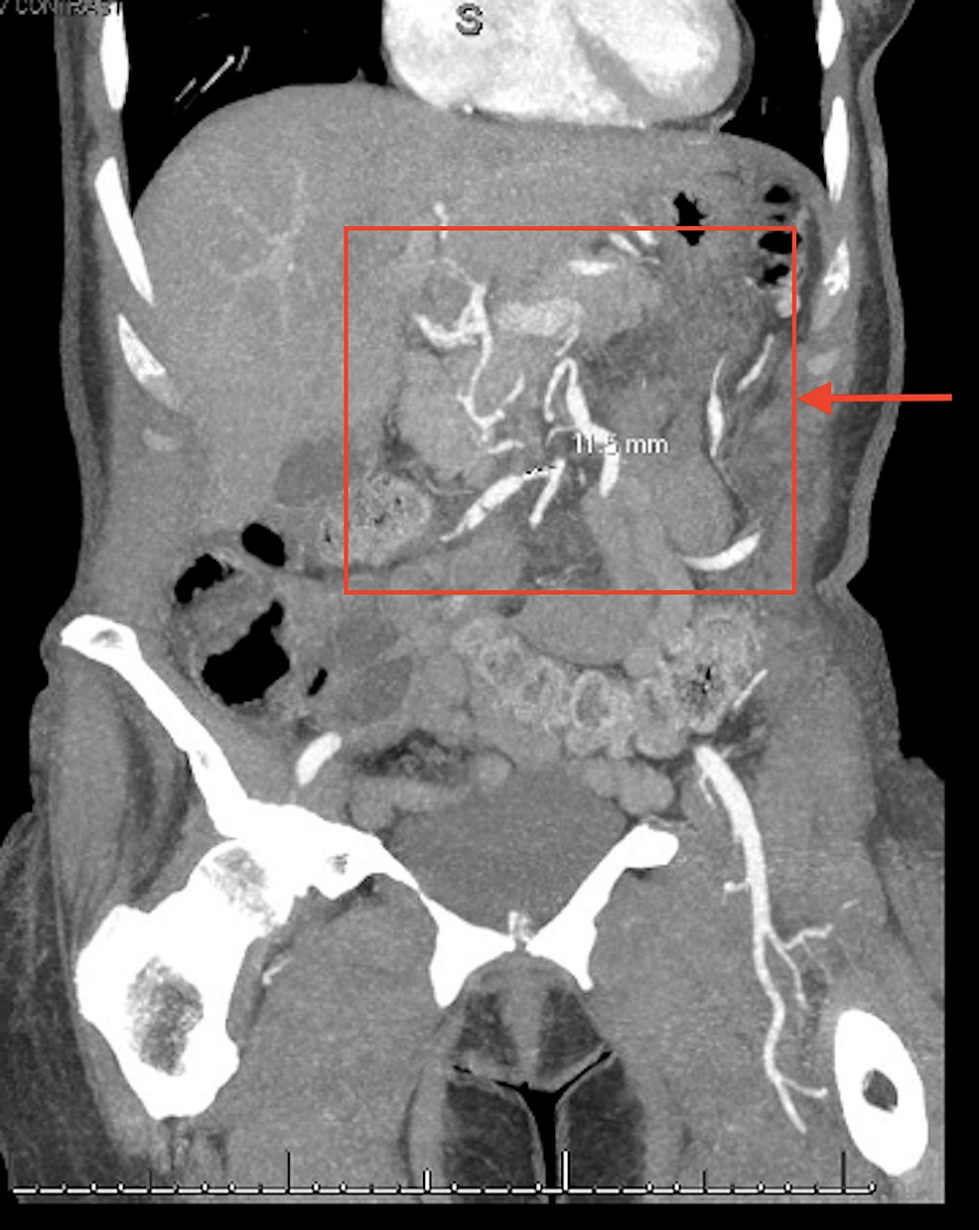
Coronal CTA demonstrating multifocal areas of SMA and IMA branch narrowing and dilation (arrow). CTA: computed tomography angiography, SMA: superior mesenteric artery, IMA: inferior mesenteric artery.

We present a case of a 66-year-old female with no significant past medical history who presented with chest pain and epigastric pain for a duration of one day. Her vital signs were within normal limits aside from blood pressure of 164/81 mmHg, and physical examination was remarkable only for abdominal pain that was out of proportion to the examination findings. Chest x-ray and computed tomography (CT) of the abdomen and pelvis were unremarkable for findings that could explain her symptoms. Subsequent CTA of the abdomen noted a celiac artery dissection (Figure 1), multifocal areas of severe vessel narrowing and dilation involving branches of the superior mesenteric artery (SMA) and inferior mesenteric artery (IMA) (Figure 2), and bilateral renal infarctions. These arterial changes were localized to the mesenteric vasculature and were originally proposed to be secondary to segmental arterial mediolysis (SAM). Therefore, she was subsequently treated with intravenous antihypertensives and balloon angioplasty of the celiac artery dissection. Despite this treatment, the patient continued to have progression of disease, leading to further vessel narrowing and new areas of aneurysmal dilation and arterial dissection. Due to the progression of the disease despite proper management for SAM, alternative diagnoses were then considered. A broad infectious disease workup, including testing for human immunodeficiency virus (HIV) and HBV, was unremarkable. In addition, the inflammatory markers ESR and CRP were significantly elevated at 60 mm/hour and 18 mg/L, respectively. Thus, the etiology began to point towards inflammatory vasculitis. Other markers for vasculitis, such as c-antineutrophilic cytoplasmic antibodies (ANCA) and p-ANCA, were negative. Once an infectious etiology was ruled out, it was considered safe to begin a trial of high-dose intravenous steroids. Within a short period of treatment, the patient responded well and was eventually deemed stable for discharge. She declined cyclophosphamide treatment and was maintained on a long taper of steroids alone. Since then, she has had no recurrence or progression of the disease.

## Discussion

Before diagnosing PAN, other possible diagnoses were excluded from the differential. The disease was confined to the mesenteric vasculature and consisted of the same pattern of arterial narrowing alternating with areas of dilation and dissection that is seen in cases of SAM [4]. SAM is usually confined to the mesenteric vasculature [4], as was our patient’s pattern of PAN. The disease process for SAM is typically controlled with blood pressure management and surgical intervention if needed [4]. Our patient did not respond to the aforementioned measures, so alternative diagnoses were considered. Fibromuscular dysplasia (FMD) was also an important diagnosis to consider. It shares some features with SAM, and is typically treated with anticoagulation and/or surgical intervention [5]. Unlike PAN, SAM and FMD are not inflammatory disorders but rather inherent defects of the arterial wall structure [4-5]. It is also important to rule out an infectious etiology in these patients, including testing for HIV and HBV [3].

Once the aforementioned etiologies were ruled out, steroids were initiated for our patient, to which she had a good response and had no further progression of disease. She was offered cyclophosphamide, which is prescribed along with steroids in cases of PAN [2]. However, she declined this treatment. Her improvement on steroids alone indicates that an underlying inflammatory process was the etiology of her mesenteric vessel disease and lessens the likelihood of other etiologies such as SAM, FMD, or active infection, all of which would not respond to this form of treatment.

PAN is classically known to not be associated with ANCA, as most other forms of vasculitis are, which helps to narrow the diagnosis of PAN [3]. It is also known to spare the lungs [1]. Both of these findings were applicable to our patient. The kidneys are also commonly involved in PAN, as evidenced by areas of renal infarction on imaging studies and reflex hypertension as a compensatory response to renal artery involvement [1], both of which were also present in our patient. The skin is one of the most commonly affected organs in PAN and serves as an easily accessible site for biopsy along with muscle and nerve tissue [1]. Biopsy will show areas of arterial wall panmural fibrinoid necrosis with polymorphonuclear cell infiltrate, and these findings can definitively diagnose PAN [6]. Our patient’s PAN was confined to the mesenteric vasculature with no cutaneous, neurologic, or musculoskeletal involvement, which is a rare anomaly for the disease. Most cases of PAN in the literature present with constitutional symptoms such as fevers, weight loss, malaise, as well as arthralgias, myalgias, and renal involvement [7]. Gastrointestinal involvement is noted to be associated with high rates of morbidity and mortality and most commonly involves the small intestine and gallbladder [7]. Gastrointestinal involvement can affect anywhere from 14% to 66% of PAN patients [7]. In our patient's case, biopsy was not ultimately performed as it was deemed that the risk associated with obtaining a tissue sample would outweigh any possible benefit. Diagnosis of PAN can still be made without a supporting biopsy, but rather with the use of other objective data such as CTA findings paired with lab findings [1], which is how our patient was diagnosed. Like most patients diagnosed with PAN, our patient was deemed as having idiopathic PAN, as no discernable etiology was ultimately found.

## Conclusions

PAN is a rare systemic vasculitis that can be definitively diagnosed with biopsy of an affected organ. However biopsy cannot always be safely performed in certain clinical settings. Nevertheless, the diagnosis can still be reasonably made with other available data. These data include specific CTA findings for PAN, elevated inflammatory markers, negative ANCA testing, and positive response to immunomodulating therapies and steroids. It is also important to rule out other vascular diseases that can present similarly to PAN, such as SAM, FMD, or infectious diseases.
